# Non-invasive left ventricular pressure-volume loops from cardiovascular magnetic resonance imaging and brachial blood pressure: validation using pressure catheter measurements

**DOI:** 10.1093/ehjimp/qyad035

**Published:** 2023-10-25

**Authors:** Per M Arvidsson, Peregrine G Green, William D Watson, Mayooran Shanmuganathan, Einar Heiberg, Giovanni Luigi De Maria, Håkan Arheden, Neil Herring, Oliver J Rider

**Affiliations:** Department of Cardiovascular Medicine, John Radcliffe Hospital, Oxford Centre for Clinical Magnetic Resonance Research, University of Oxford, Oxford OX3 9DU, United Kingdom; Oxford Heart Centre, John Radcliffe Hospital, Oxford OX3 9DU, United Kingdom; Department of Physiology, Anatomy and Genetics, University of Oxford, Oxford OX1 3PT, United Kingdom; Department of Cardiovascular Medicine, John Radcliffe Hospital, Oxford Centre for Clinical Magnetic Resonance Research, University of Oxford, Oxford OX3 9DU, United Kingdom; Department of Cardiovascular Medicine, Heart and Lung Research Institute, Papworth Road, Cambridge CB2 0AY, United Kingdom; Department of Cardiovascular Medicine, John Radcliffe Hospital, Oxford Centre for Clinical Magnetic Resonance Research, University of Oxford, Oxford OX3 9DU, United Kingdom; Cardiology Department, Buckinghamshire Healthcare NHS Trust, Wycombe Hospital, Queen Alexandra Road, High Wycombe HP11 2TT, United Kingdom; Heart Transplant Department, Harefield Hospital, Royal Brompton and Harefield Hospitals, Hill End Road, Harefield UB9 6JH, United Kingdom; Clinical Physiology, Department of Clinical Sciences Lund, Lund University, Skåne University Hospital, Lund, Sweden; Oxford Heart Centre, John Radcliffe Hospital, Oxford OX3 9DU, United Kingdom; Clinical Physiology, Department of Clinical Sciences Lund, Lund University, Skåne University Hospital, Lund, Sweden; Department of Physiology, Anatomy and Genetics, University of Oxford, Oxford OX1 3PT, United Kingdom; Department of Cardiovascular Medicine, John Radcliffe Hospital, Oxford Centre for Clinical Magnetic Resonance Research, University of Oxford, Oxford OX3 9DU, United Kingdom

**Keywords:** cardiovascular magnetic resonance imaging, pressure-volume loops, haemodynamics, heart failure, physiology, non-invasive

## Abstract

**Aims:**

Left ventricular (LV) pressure-volume (PV) loops provide gold-standard physiological information but require invasive measurements of ventricular intracavity pressure, limiting clinical and research applications. A non-invasive method for the computation of PV loops from magnetic resonance imaging and brachial cuff blood pressure has recently been proposed. Here we evaluated the fidelity of the non-invasive PV algorithm against invasive LV pressures in humans.

**Methods and results:**

Four heart failure patients with EF < 35% and LV dyssynchrony underwent cardiovascular magnetic resonance (CMR) imaging and subsequent LV catheterization with sequential administration of two different intravenous metabolic substrate infusions (insulin/dextrose and lipid emulsion), producing eight datasets at different haemodynamic states. Pressure-volume loops were computed from CMR volumes combined with (i) a time-varying elastance function scaled to brachial blood pressure and temporally stretched to match volume data, or (ii) invasive pressures averaged from 19 to 30 sampled beats. Method comparison was conducted using linear regression and Bland-Altman analysis. Non-invasively derived PV loop parameters demonstrated high correlation and low bias when compared to invasive data for stroke work (R^2^ = 0.96, *P* < 0.0001, bias 4.6%), potential energy (R^2^ = 0.83, *P* = 0.001, bias 1.5%), end-systolic pressure-volume relationship (R^2^ = 0.89, *P* = 0.0004, bias 5.8%), ventricular efficiency (R^2^ = 0.98, *P* < 0.0001, bias 0.8%), arterial elastance (R^2^ = 0.88, *P* = 0.0006, bias −8.0%), mean external power (R^2^ = 0.92, *P* = 0.0002, bias 4.4%), and energy per ejected volume (R^2^ = 0.89, *P* = 0.0001, bias 3.7%). Variations in estimated end-diastolic pressure did not significantly affect results (*P* > 0.05 for all). Intraobserver analysis after one year demonstrated 0.9–3.4% bias for LV volumetry and 0.2–5.4% for PV loop-derived parameters.

**Conclusion:**

Pressure-volume loops can be precisely and accurately computed from CMR imaging and brachial cuff blood pressure in humans.

## Introduction

Left ventricular (LV) pressure-volume (PV) loops contain a wealth of information with the potential to improve assessment of cardiac health, providing decision-making support for clinicians and valuable data for researchers.^1^ While cardiovascular magnetic resonance (CMR) imaging is the gold-standard for accurate assessment of cardiac volumes, load-independent contractile performance and other aspects of ventricular dynamics require knowledge of intracavity pressure currently only obtainable through LV catheterization. Current guidelines for the management of suspected heart failure recommend CMR for assessment of myocardial structure and function^2^ but also acknowledge the need for improved assessment of patient-specific haemodynamic measures, including left heart catheterization.

Due to the invasive nature of conductance catheter experiments, PV loop analysis is not conducted routinely in clinical practice. Ethical considerations such as risk/benefit and procedural costs further limit PV loop applications. Furthermore, while LV catheterization is the only way to obtain absolute pressures, ventricular volumes are most accurately captured by short-axis CMR image acquisition. As such, whilst providing gold-standard haemodynamic assessment, PV loop interrogation does not provide the best quality volumetric assessment. Should non-invasive measures accurately predict invasive PV loops, the inherent risk and expense of invasive LV catheterization could then be avoided.

**Figure 1:**
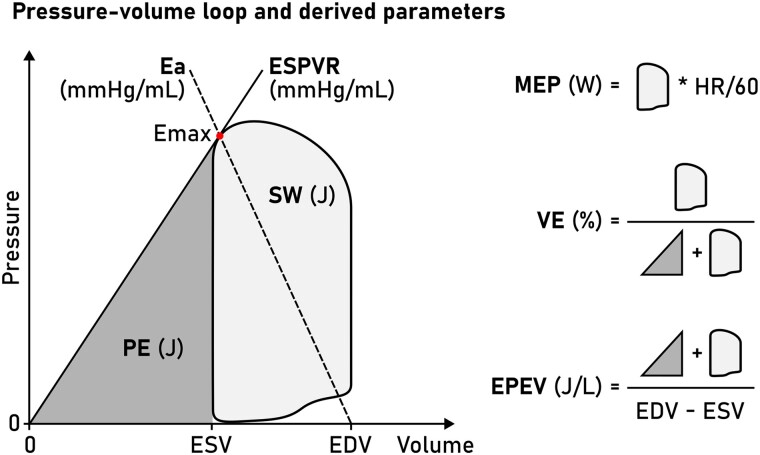
Schematic LV PV loop. A PV loop is inscribed between the EDV and ESV. The point of maximal elastance (E_max_, red dot) is used to compute the ESPVR (solid line, slope from V_0_, here simplified to 0 mL) and the arterial elastance function (E_a_, dashed line, slope to pressure = 0 at EDV). The area enclosed by the PV loop is the SW, and the area enclosed by the ESPVR and ESV (grey triangle) is the PE. These measures enable subsequent calculation of MEP, VE, and EPEV.

Recent development has seen the introduction of non-invasively computed PV loops from CMR volumetry and a brachial blood pressure measurement,^3^ combining LV volumes with a time-varying elastance function to compute time-resolved LV pressures.^4^ The advantage of this method is that it can be widely implemented using standard CMR images and provides measures of myocardial stroke work (SW), potential energy (PE), end-systolic pressure-volume relationship (ESPVR) (inotropic state), energy per ejected volume (EPEV), ventricular efficiency (VE), arterial elastance, and mean external power (MEP) (*Figure 1*). While the method has been validated using invasive data from a porcine model^3^ and sensitivity tested using a dobutamine infusion in healthy humans,^5^ it remains to be validated in patients using invasive LV pressure recordings from PV loops.

The aim of this project was therefore to provide the first validation of non-invasively computed PV loops against invasive measures in humans, testing the hypothesis that the methods are interchangeable.

## Methods

### Patient recruitment

This was a *post hoc* analysis of data from a study designed to investigate the effects of metabolic substrate switching on myocardial function in heart failure.^6^ Patients were originally recruited from the cardiology services of the Oxford University Hospitals NHS Foundation Trust. All participants gave written informed consent. The study was conducted in accordance with the Helsinki declaration and was approved by the National Research Ethics Service (REC reference 18/SC/0170). OJR had full access to all the data and took responsibility for its integrity and data analysis.

Inclusion criteria were as follows: patients at least 18 years of age, willing and able to give informed consent, and diagnosed with non-ischaemic heart failure with reduced ejection fraction (HFrEF) meeting criteria for cardiac resynchronization therapy (CRT) as per the 2016 European Society of Cardiology guidelines.^7^ All patients were scheduled for CRT implantation as part of routine clinical care, and all procedures were in accordance with institutional guidelines. After completing all study procedures, each dataset was assessed for technical quality and completeness. Only datasets where complete imaging and invasive data were available, and which were acquired during sinus rhythm, were considered for inclusion.

Exclusion criteria included inability to safely undergo study procedures, general MRI safety criteria (implanted metal clips, metal shrapnel in body or metal fragments in eye, history of severe claustrophobia) as well as disturbances of normal fat metabolism and allergies to soya or egg. Patients were also screened and excluded for severe liver damage, tuberculosis, pulmonary disease, anaemia, coagulation disorders, and recent fractures of pelvis or long bones.

### Experimental protocol

**Figure 2:**
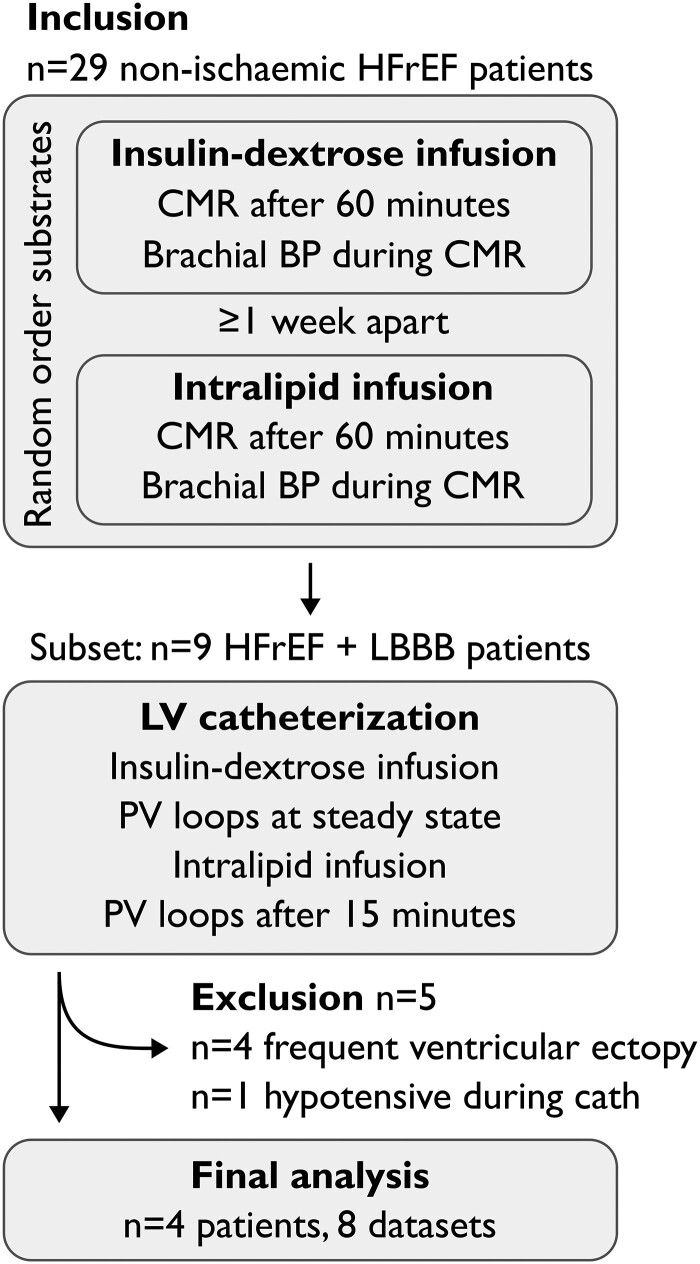
Study protocol.

Study participants underwent three study visits, summarized in *Figure 2*. On the first visit, participants were randomized to receive either insulin/dextrose infusion or lipid emulsion (Intralipid™, Fresenius Kabi, UK) infusion after fasting overnight, with the other infusion given on the second visit at least seven days later, again after fasting overnight. The third visit consisted of the CRT implantation. Participants were fasted overnight as per clinical protocol and started on an insulin/dextrose infusion before the implant. During the implant, a pressure-conductance catheter was placed in the LV. Following the recording of pressure and conductance data, participants were switched onto an Intralipid™ infusion, with PV loops recorded again after 15 min of infusion.

### Insulin/dextrose infusion protocol

A standard mid-physiological range euglycaemic hyperinsulaemic clamp was used, with participants clamped at their fasting blood glucose level, using a continuous infusion of insulin (Actrapid, Novo, Denmark) at 0.8 mU/kg/min following a 10-minute loading regime and variable rates of dextrose infusion (200 mg/mL). Venous blood glucose measurements were performed every 5 min for the first hour to ensure a steady state had been reached. CMR was then performed (total protocol length approx. 1 h), and brachial blood pressure was measured once near the end of the protocol (Expression^TM^ MR200 MRI safe monitoring system, MR Devices, UK), less than 1 h after acquiring short-axis images.

### Intralipid infusion protocol

To elevate free fatty acid levels, a continuous infusion of Intralipid™ 200 mg/mL was given at 60 mL/h. In patients not on anticoagulant medications, unfractionated heparin (Monoparin, CP Pharmaceuticals, UK, or equivalent) was also infused at 0.4 U/kg/min to further increase triglyceride breakdown. During invasive measurements, heparin was given as an intra-arterial bolus to keep activated clotting time ≥250 ms. CMR imaging was commenced at a steady state 60 min after starting Intralipid infusion, and brachial blood pressure was measured once near the end of the protocol, within one hour after short-axis imaging.

### LV catheterization

An Inca™ conductance catheter (CD Leycom, NL) was passed via 8Fr femoral arterial access to the LV apex, following calibration to atmospheric pressure (‘zeroing’) prior to implant. PV loops were recorded for 1 min at a sampling frequency of 250 Hz. The largest measured volume was equalized to the end-diastolic volume measured from the preceding CMR. Conductance catheter data was analysed using commercial software provided by the manufacturer (Conduct NT™, version 3.18.1, CD Leycom, NL). A 10 Hz filter was applied to recordings. The pressure was adjusted to correct for any offset recorded at the time of catheter withdrawal from the femoral artery. Any catheter segments not in the LV were discarded from the final calculations and auto systolic and diastolic markers were manually checked for accuracy and adjusted if necessary. In the case of ectopy, the ectopic beat and the preceding and following beats were discarded from the recording. Frequent ectopy was grounds for exclusion.

### Cardiac magnetic resonance imaging

Study participants underwent CMR imaging in the supine position at 3T (Magnetom Trio, Siemens Healthcare, Erlingen, Germany) using a cardiac coil. Balanced steady-state free precession (bSSFP) imaging was performed in the horizontal and vertical long-axis projections and a short-axis stack covering the entire left ventricle. Typical imaging parameters were: echo time (TE)/repetition time (TR) 1.45/46 ms, flip angle 50°, in-plane resolution 1.6 × 1.6 mm, slice thickness 8 mm, no slice gap. Images were acquired during end-expiratory breath holds. Retrospective ECG gating was employed and images were reconstructed to 25 frames per cardiac cycle.

### Image analysis

Image analysis was conducted using Segment 4.0 R11026 (Medviso, Lund, Sweden). LV volumes were computed over the cardiac cycle from time-resolved delineations of the LV endocardial boundaries in the short-axis stack, as previously described.^8^ Image segmentation was repeated after one year to provide intraobserver data with the reader blinded to previous results. Interobserver analysis was similarly conducted for cardiac volumes.

### Computation of PV loops from CMR and brachial blood pressure

PV loops were computed using a previously published algorithm^3,5^ trained on invasive data from porcine experiments. In short, the algorithm uses a digitized time-varying elastance curve^4^ to model dynamic LV pressures coupled to LV volumes measured in CMR images. The elastance model was scaled in time so that the minimal LV volume (end-systolic timeframe in CMR images) coincided with the middle of the elastance curve downslope. The elastance curve was scaled in amplitude to match LV peak pressure (LVP_systole_) and LV end-diastolic pressure (EDP). An approximative LVP_systole_ was calculated from brachial pressure according to a previously proposed expression,^9^


$${\mathrm{LVP}}_{\mathrm{systole}}=2/3\;\mathrm{SBP}+1/3\;\mathrm{DBP},$$

where SBP is systolic blood pressure and DBP is diastolic blood pressure. Since EDP is not possible to derive from a brachial blood pressure measurement, the user is prompted to estimate the EDP. A previous sensitivity analysis found varying user-input EDP in the 0–15 mmHg range had little effect on PV-loop-derived metrics in a porcine model.^3^ Data shown in this manuscript result from setting EDP to 7.5 mmHg for all patients. A sensitivity analysis for EDP estimates of 3–40 mmHg was also conducted, details are below.

Ventricular pressure *P(t)* can be computed as


$$P(t)=E(t)\times (V(t)\;\text{–}\;V_{0}),$$

where *E(t)* is the time-varying elastance and V_0_ is the intercept of the ESPVR and the volume axis, i.e. the volume where LV pressure would be 0 mmHg. Setting V_0_ to zero^10,11^ simplifies the expression to:


P(t)=E(t)×V(t),

which was computed and interpolated to 100 points per cardiac cycle, and subsequently plotted against *V(t)* to visualize PV loops.

### Computation of PV loops from CMR and invasive pressure

Reference PV loops were computed using LV pressure curves averaged from 19 to 30 recorded cardiac cycles. Pressure curves from individual cycles were resampled to 250 points on a reference time axis using linear interpolation and subsequently averaged to produce a single pressure-time curve *P(t)*.

Similar to the non-invasive approach, we used CMR LV volumes to plot *P(t)* vs. *V(t)*. This enables a direct validation of the pressure modelling employed in the non-invasive method and disregards the methodological differences between CMR and conductance catheter experiments for volumetry.

### Definitions of PV loop metrics

We studied seven PV loop-derived metrics, summarized in *Figure 1*. Myocardial SW is a measure of the external work performed by the ventricle in ejecting blood and was defined as the area inscribed by the PV loop. The ESPVR was defined as the slope between V_0_, set to zero, and E_max_, which is the point where the time-varying elastance function reaches its highest value (maximal myocardial stiffness) and hence a proxy measure of contractility.

The mechanical PE is the internal energy to overcome in order to eject blood.^1^ It is optimally measured as the area inscribed by the (nonlinear) end-systolic and EDP-volume relationships below the end-systolic volume (ESV). Here we simplified PE and defined it as the area of a triangle with a base between V_0_ and ESV, and height equal to the ESPVR at ESV. The total mechanical energy of each cardiac cycle is defined as the entire pressure-volume area (PVA), equal to SW + PE, and is approximately proportional to myocardial oxygen consumption.^12^ VE was computed as SW/PVA.

The MEP delivered by the ventricle was calculated as SW*HR (Heart Rate)/60. EPEV is a measure of the energy spent ejecting a stroke volume (SV) and was computed as PVA/SV. Finally, the arterial elastance (E_a_) was computed as the slope from E_max_ to the point of end-diastolic volume and zero pressure, equal to E_max_/SV.

### Sensitivity analysis for different estimations of EDP

The effects of different estimated EDP values on SW, VE, MEP, and EPEV were assessed. PV loops were computed with EDP set to 3, 7.5, 10, 16, 25, and 40 mmHg to reflect an extremely wide range of possible filling pressures. We also evaluated in each individual case the effect of using the patient-specific invasive EDP compared to assuming an EDP of 7.5 mmHg.

### Statistical analysis

Statistical analysis was conducted using GraphPad Prism 9.5.0 (GraphPad Software, La Jolla, USA). Method agreement between invasive and CMR metrics as well as intraobserver and interobserver variability was assessed using linear regression and Bland-Altman analysis and presented as bias with limits of agreement (LoA).^13^ For the interobserver study we also calculated the intraclass correlation coefficient for absolute agreement using a two-way random effects model. Paired *t-*tests were used to examine intraindividual variability between scan sessions and using different parameters for computation of PV loops. Statistical significance was assigned at the *P* < 0.05 level.

## Results

### Population characteristics

Out of the 29 patients participating in the parent study, in addition to CMR, nine had undergone LV catheterization with attempted PV recording during metabolic substrate infusion (*Figure 2*). Of these, four patients were excluded due to high ectopic burden. A total of nine studies from five patients had LV pressure recordings of adequate quality for validation purposes. One patient (one dataset) was then excluded due to having been significantly hypotensive during the catheterization procedure, which resulted in a large difference between invasive and brachial blood pressures.

Four patients (one woman) with average age of 63 (range 58–69) were included in the final analysis. All patients were in heart failure with left bundle branch block and echocardiographic EF <35% despite optimal medical therapy. Patient characteristics are summarized in *Table 1*.

**Table 1:** Baseline subject characteristics

	Subject 1	Subject 2	Subject 3	Subject 4
Sex	M	M	F	M
Age (years)	79	69	64	60
Height (m)	1.82	1.82	1.69	1.9
Weight (kg)	73	99	99	94
Body mass index (kg/m^2^)	22.0	29.9	34.6	26.0
Body surface area (m^2^)	1.92	2.24	2.16	2.23
LV ejection fraction (%)	22	33	31	37
NT-proBNP (pg/mL)		3268	1430	
BNP (pg/mL)	723			216
Resting blood pressure (mmHg)	100/61	156/95	105/56	110/65
ECG rhythm	Sinus	Sinus	Sinus	Sinus
Beta blocker	1	1	1	1
ACEi/ARNi	1	1	1	1
Aldosterone inhibitor	1	0	1	1
SGLT2 inhibitor	0	0	0	0
Diuretics	1	0	1	1

LV, left ventricle; NT-proBNP, N-terminal pro B-type natriuretic peptide; BNP, B-type natriuretic peptide; ACEi, angiotensin converting enzyme inhibitor; ARNi, angiotensin II receptor/neprilysin inhibitor; SGLT2, sodium/glucose transport protein 2.

### Haemodynamic parameters and blood pressure modelling

Haemodynamic and volumetric parameters are summarized in *Table 2*. Heart rate was similar between catheter procedures and CMR examinations as well as between the two CMR examinations. Likewise, brachial blood pressure was similar between substrates for systolic and diastolic pressure. Brachial SBP measured at CMR correlated with invasive peak pressures (R^2^ = 0.64, *P* = 0.016) with an average positive bias of 11 mmHg, reflecting the narrowing of the pulse wave in the vasculature. Within each individual LV pressure recording, the peak systolic pressure varied on average by ±6.1 mmHg (1.96 SD).

**Table 2:** Haemodynamic and volumetric parameters

	CMR			Catheterization			CMR vs. cath	
	I + D	Lipid	*P* value	I + D	Lipid	*P* value	*P* value I + D	*P* value Lipid
Brachial BP (mmHg)	122/65	123/65	0.68/0.74					
Peak systolic/EDP (mmHg)				107/12	112/11	0.17/0.61		
Heart rate (bpm)	70	72	0.35	75	76	0.72	0.31	0.42
End-diastolic volume (mL)	336	324	0.22					
ESV (mL)	244	221	0.23					
Ejection fraction (%)	28	32	0.28					

Average values. I + D, insulin + dextrose; Lipid, Intralipid™ infusion.

### PV loop metrics

**Figure 3:**
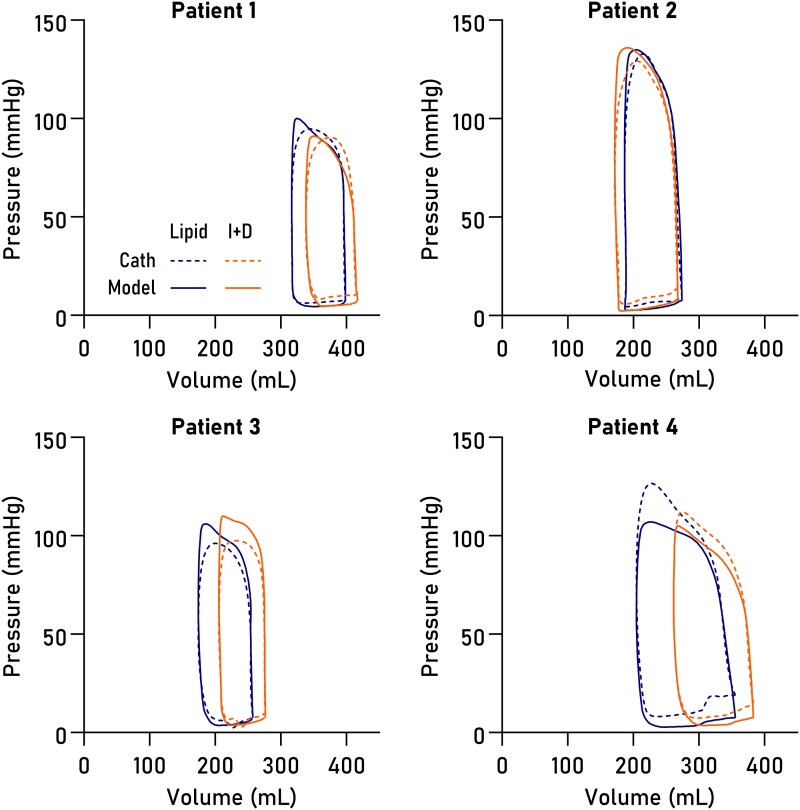
PV loops. Loops in eight datasets from four patients, computed from CMR volumetry data paired with pressures modelled from time-varying elastance (solid lines) or pressures from LV catheterization (dashed lines), on intravenous Intralipid™ (lipid, blue) or insulin and dextrose infusion (I + D, orange).

**Figure 4:**
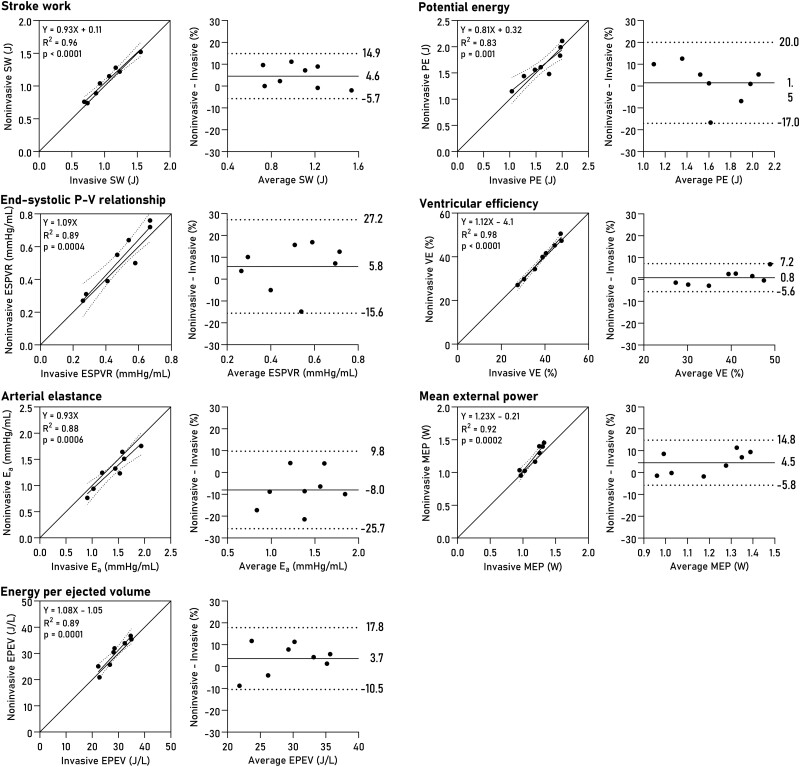
PV loop-derived parameters. Linear regression and Bland-Altman plots show bias and limits of agreement. SW, stroke work; PE, potential energy; ESPVR, end-systolic pressure-volume relationship; VE, ventricular efficiency; E_a_, effective arterial elastance; MEP, mean external power; EPEV, energy per ejected volume.

Non-invasively derived PV loops were computed for all datasets and are shown together with invasive loops in *Figure 3*. Loop-derived parameters (*Figure 4*) demonstrated high correlation and low bias when compared to invasive data for SW (R^2^ = 0.96, bias 4.6%, LoA −5.7–14.9%, *P* < 0.0001), PE (R^2^ = 0.83, *P* = 0.001, bias 1.5%, LoA −17.0–20.0%), ESPVR (R^2^ = 0.89, *P* = 0.0004, bias 5.8%, LoA −15.6–27.2%), VE (R^2^ = 0.98, *P* < 0.0001, bias 0.8%, LoA −5.6–7.2%), arterial elastance (R^2^ = 0.88, *P* = 0.0006, bias −8.0%, LoA −25.7–9.8%), MEP (R^2^ = 0.92, *P* = 0.0002, bias 4.4%, LoA −5.8–14.8%), and EPEV (R^2^ = 0.89, *P* = 0.0001, bias 3.7%, LoA −10.5–17.8%).

### Intraobserver and interobserver variability

**Figure 5:**
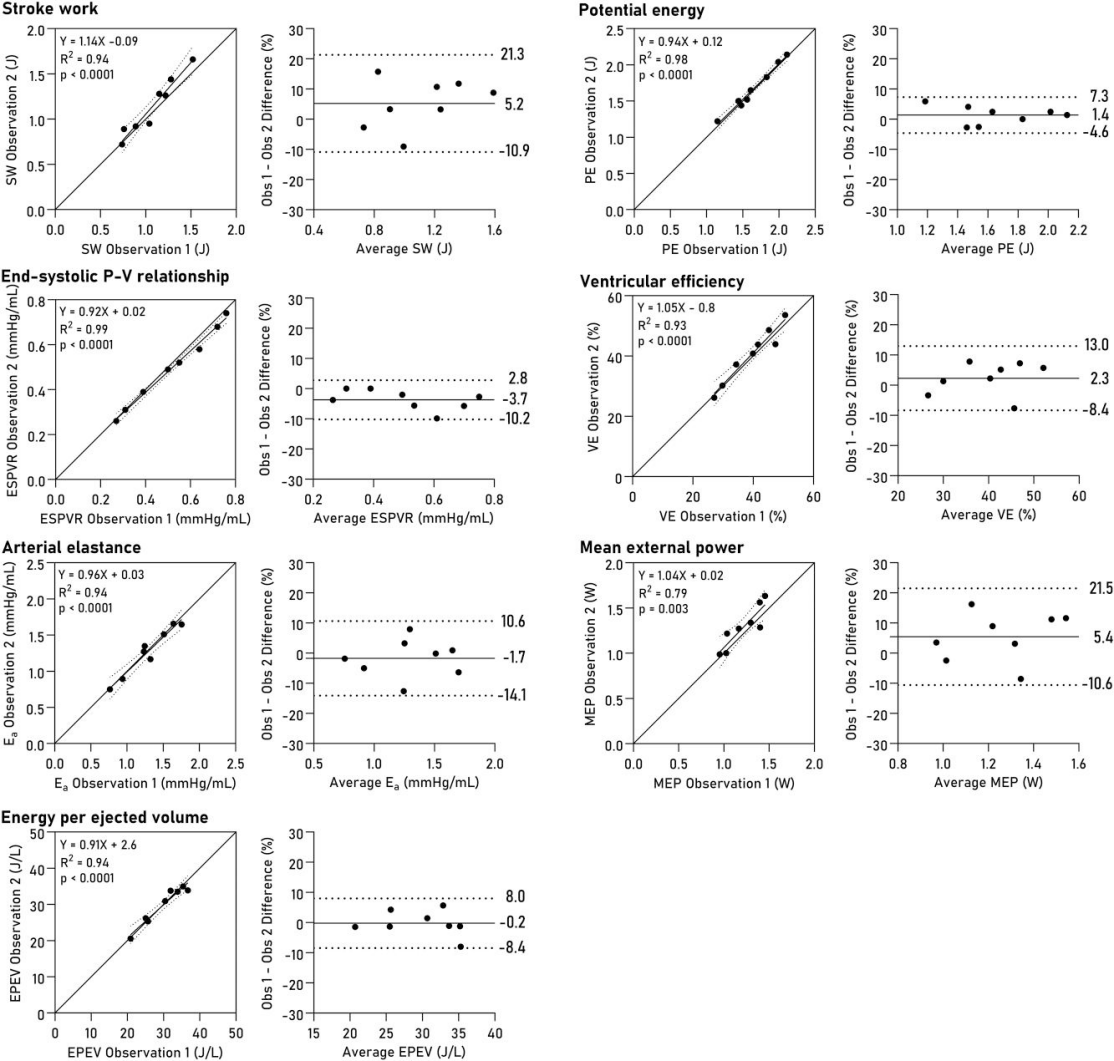
Intraobserver variability for PV loop parameters. Linear regression and Bland–Altman plots show bias and limits of agreement. SW, stroke work; PE, potential energy; ESPVR, end-systolic pressure-volume relationship; VE, ventricular efficiency; E_a_, effective arterial elastance; MEP, mean external power; EPEV, energy per ejected volume.

Intraobserver variability was assessed by repeating LV delineations after one year with the observer blinded to previous results. The average volumetric bias was 2% (7 mL) for end-diastolic volume, 1% (2 mL) for ESV, and 5% (5 mL) for SV (see Supplementary data online, *Figure S1*). The intraobserver analysis for loop-derived parameters is summarized in *Figure 5*. The bias for SW was 5.2% (LoA −10.9–21.3), for PE 1.4% (LoA −4.6–7.3), for ESPVR −3.7 (LoA −10.2–2.8), for VE 2.3% (LoA −8.4–13.0), for arterial elastance −1.7% (LoA −14.1–10.6), for MEP 5.4% (LoA −10.6–21.5), and for EPEV −0.2% (LoA −8.4–8.0).

We similarly assessed interobserver variability for volumetry, shown in Supplementary data online, *Figure S2*. Bias was on average 0.6% (0.5 mL) for end-diastolic volume, 3% (4.5 mL) for ESV, and −4.5% (4 mL) for SV. The intraclass correlation coefficient was 0.94–0.99 (Excellent reliability).

### Sensitivity to variations in EDP

**Figure 6:**
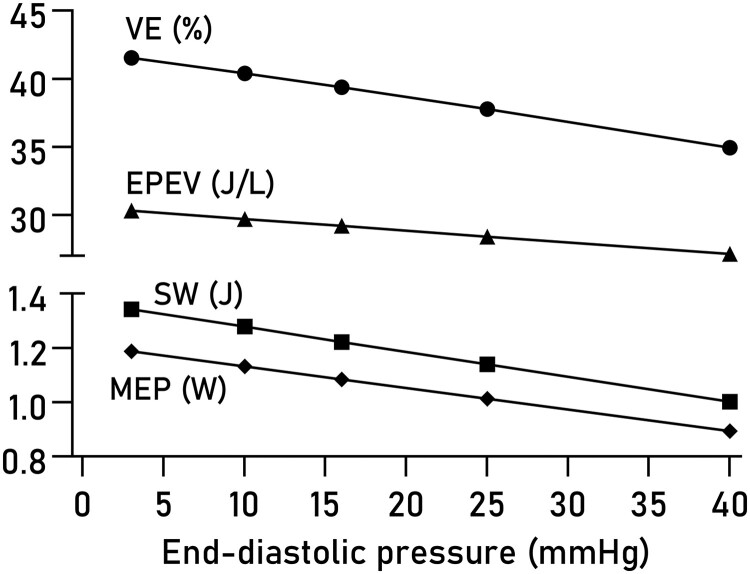
Effect of varying EDP on PV loop parameters. The average effect of varying the user-estimated EDP is shown for VE, EPEV, SW, and MEP.

**Figure 7:**
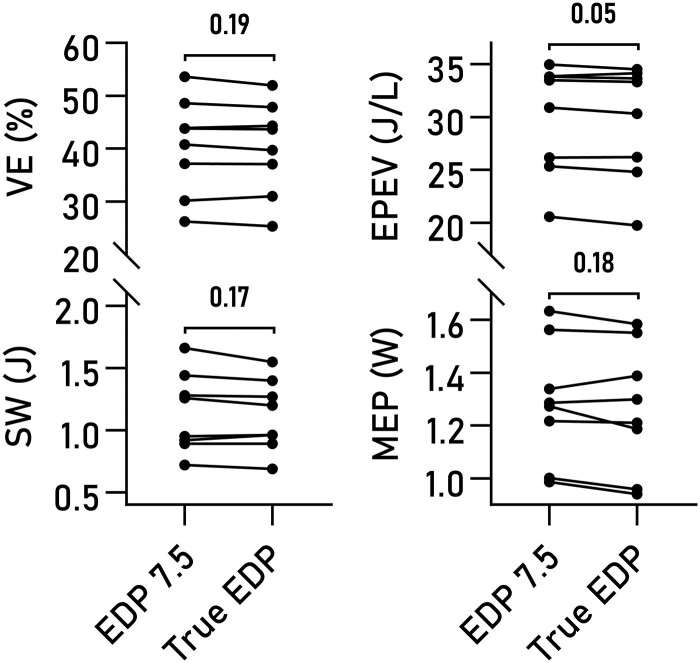
Estimated and actual end-diastolic pressures. Pairwise comparison of VE, SW, MEP, and EPEV, computed using an assumed EDP of 7.5 mmHg compared to using the true value from invasive measurements. No significant differences were seen.

Varying EDP within a physiological range of values had minor effects on computed VE, EPEV, MEP, and SW (*Figure 6*). The effect of using true measures compared to a fixed EDP of 7.5 mmHg was not statistically significant on any of the studied parameters (*Figure 7*), although a trend was seen for EPEV.

## Discussion

In this paper, we present the first human validation of non-invasive PV loops from CMR using invasive LV pressure recordings. Our findings indicate that non-invasive PV loops are robust, as they make efficient use of the accurate and precise volumetry of CMR combined with a method for estimating intraluminal LV pressure dynamics from a simple brachial blood pressure measurement. All evaluated parameters displayed good agreement between invasive and non-invasive approaches, combining sensitivity to changes in haemodynamic state with robustness to variations in user-estimated EDP. These findings suggest that non-invasive PV loop analysis is ready for application in intervention and population studies in selected patient groups. Loop-derived parameters such as cardiac efficiency, SW, and contractility hold potential as possible therapeutic targets for personalized medicine.

### Patient selection and generalizability of results

We opted to study a population of heart failure patients meeting current criteria for CRT not only to avoid catheterization of patients with no clinical indication for an invasive procedure but also to confirm the validity of these measures in patients with abnormal LV geometry. Conductance catheter studies in patients with LV dyssynchrony are often complicated by the irregular, rocking motion of the myocardium, which negatively affects the quality of the volume estimation. While the pressure recordings in our patient cohort were of good quality throughout (exclusion was primarily due to high ectopic burden), only four conductance datasets were deemed technically adequate (data not shown), and direct comparison between fully non-invasive and fully invasive PV loops was therefore not possible with preserved statistical power.

Whilst dyssynchronous contraction is seen on CMR imaging, segmentation of short-axis cine images acquired using standard ECG gating produced PV loops with physiological shapes and good fidelity compared to invasive pressures. As such, we believe this algorithm is likely to perform at least as well in subjects with normal LV function and electromechanical synchrony. Furthermore, this study demonstrates that non-invasive PV loop analysis is feasible with good accuracy and precision even in patients where invasive experiments may not render data of sufficient quality. Indeed, the variability of the resulting PV loop parameters was similar in magnitude to the beat-to-beat variability seen in the invasive pressure recordings. The method thereby expands PV loop analysis to both low-risk patients and healthy controls, where the risk associated with invasive procedures may not be ethically justifiable, and to patients with severely impaired ventricular function, where accurate volumes may be difficult to obtain using conductance measurements. With the exception of significant valvular disease, the results from this study, therefore, support wide application of non-invasive PV loops.

### Sources of error for contractility and arterial elastance

Arterial elastance, E_a_, is a measure approximating the resistance in the peripheral vasculature. As such it is dependent on total peripheral resistance, which is unknown, and heart rate, which was similar between the catheterization and CMR experiments. It also depends on E_max_, which is defined as the point along the PV loop of maximal ventricular stiffness. In the literature E_max_ is sometimes used interchangeably with end-systolic stiffness, E_es_, although the two are not technically the same; maximal stiffness typically occurs sometime before cessation of ejection.^5,9,14^

**Figure 8:**
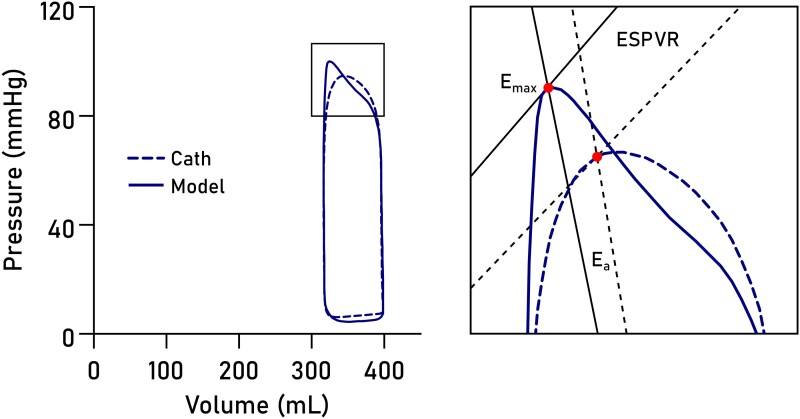
Sources of error for approximation of contractility and arterial elastance. The point of maximal elastance, E_max_ (red dot), is pivotal for determining both ESPVR and arterial elastance (E_a_) slopes. Errors in E_max_ timing may arise from imperfect temporal scaling of the elastance function. The result is a slight overestimation of ESPVR and an underestimation of E_a_ in the model (solid lines) compared to invasive data (dashed lines).

The exact timing of the modelled E_max_ will depend on the temporal scaling of the time-varying elastance function and is therefore subject to user-defined markers for end diastole and end systole. The presence of dyssynchrony complicates the determination of end systole and hence of E_max_, as postsystolic contraction prolongs the isovolumic relaxation period, subsequently affecting the temporal scaling of the time-varying elastance function. From *Figure 3*, it is evident that the upper left corners of the loops differ slightly between the invasive and modelled loops. This likely explains both the slight overestimation of ESPVR and the slight underestimation of E_a_ in the model, as illustrated in *Figure 8*.

The precise degree to which the E_max_ timing issue explains the errors in estimated E_a_ and ESPVR, in contrast to afterload conditions being truly different between CMR and cath procedures, remains to be investigated. To address this outstanding question would require CMR with simultaneous invasive and brachial pressure measurements, which was unfortunately not available at our centre.

### Clinical utility of non-invasive PV loops

PV data can be used to derive valuable information about cardiac work,^9,15–18^ and may be used to assess the effect of pharmaceutical, surgical, or device interventions.^19^ Sjöberg et al. studied the effect of dobutamine stress on LV PV relations in healthy controls and demonstrated the method’s sensitivity to changes associated with increased cardiac workload.^5^ Furthermore, we recently employed non-invasive PV loops to demonstrate that metabolic substrate switching in non-ischaemic cardiomyopathy elicits significant changes in SW and inotropic state, accompanying changes to ejection fraction, SV, and pressure change rates.^6^ Binka et al. used non-invasive PV loops to evaluate the haemodynamic effects of pulmonary valve replacement in tetralogy of Fallot and demonstrated a postoperative decrease in right ventricular SW and external power despite no improvements in LV function.^20^ And finally, Berg et al. found evidence of cardioprotective effects of hypothermia by studying ventricular-arterial coupling and VE in a porcine infarct-reperfusion model.^21^

Together, these findings indicate a potential for PV loop analysis for online evaluation and optimization of treatment strategies. For example, PV loops may be used to analyse cardiac workload immediately after initiation of resynchronization therapy to evaluate the acute effects of pacemaker programming and lead placement. The concept can be extended to the regional level using force-length loops.^19^ In contrast to invasive PV loops, which are often difficult to obtain with adequate quality in the setting of dyssynchrony,^22,23^ the method presented herein consistently performs well in the presence of significant regional wall motion abnormalities.

Further, PV loops could be implemented as an in-line monitoring solution on the CMR system to provide immediate haemodynamic feedback during pharmacological or exercise studies,^24,25^ using automated LV segmentation and input from blood pressure monitoring equipment. As the PV area is an indicator of cardiac oxygen consumption,^1^ there is an opportunity to use PV loops as a window into ventricular energetics.^26^ As long as the elastance function is known, the method would also be applicable to the quantification of atrial function.

### Diastolic pressure estimation

We found small effects of varying estimated end-diastolic pressures from 3 mmHg (very low) to 40 mmHg (severely elevated), which expands on the previous results reported by Seemann et al. who evaluated effects of estimating EDP between 0 and 15 mmHg.^3^ An EDP exceeding 16 mmHg at rest or stress is a commonly used criterion to diagnose heart failure with preserved ejection fraction.^27^ While the method is intrinsically insensitive to elevated EDP, these findings suggest it can be used to reliably quantify systolic and metabolic properties of the heart even in the presence of moderate relaxation abnormalities. Expanding the model to accurately estimate diastolic pressures would be of great clinical utility.^28,29^

### Limitations

We studied heart failure patients with LV dyssynchrony, which introduces additional challenges for conventional conductance catheter measurements through the irregular, rocking motion of the LV.^23^ The situation is further compounded by the presence of postsystolic contractions and relaxation abnormalities. Conductance catheter estimations of LV volumes presuppose volumetric calibration against an external standard, either using a known volume of saline in a beaker or optimally LV volumes from CMR. Even when meticulously calibrated, conductance catheter volumetry is plagued by nonlinearity and bias.^30–32^ In our setup, conductance-based LV volumetry was unreliable in some participants due to profound dyssynchrony and mechanical interference between the septum and the catheter, and in datasets with acceptable data quality, the normalization to CMR volumes would render a direct comparison between fully invasive and fully non-invasive PV loops methodologically unsound. CMR imaging is widely considered the reference standard for quantification of cardiac volumes. We therefore focused on validating the non-invasive pressure estimation function.

Measurement of brachial blood pressure is potentially sensitive to premature ectopic beats due to increased preload and SV in the succeeding beat, potentially leading to a slight overestimation of systolic blood pressure (BP) and hence average ESPVR. Ectopics are likely in distended and failing ventricles, and care should be taken to measure blood pressure sufficiently slowly so as to minimize the influence of breakthrough beats. As a result of the design of the parent study, in our protocol brachial BP measurement and short-axis imaging were separated by approximately 45 min. It is possible that greater accuracy could be attained through simultaneous BP and image acquisition.

The pulse pressure may be overamplified in peripheral arterial disease, resulting in an overestimation of systolic LV pressures. Conversely, in the setting of severe aortic stenosis the ventriculo-aortic pressure gradient will not be accounted for, meaning PV loops will underestimate systolic LV pressures. Neither of these settings was studied in the present work, and careful consideration is advised before implementing non-invasive PV loop analysis in the presence of significant vascular or valvular abnormalities.

## Conclusion

PV loop analysis computed from standard cine CMR imaging and brachial cuff blood pressures is precise and accurate provides non-invasive access to unique physiological information and can be readily implemented in research applications to monitor outcome parameters or search for therapeutic targets.

## Supplementary Material

qyad035_Supplementary_Data

## Data Availability

The data underlying this article cannot be shared publicly due to limitations in ethical permits. Anonymized data may be shared on reasonable request to the corresponding author.

## Lead author biography

Per M. Arvidsson currently serves as postdoctoral research fellow at the Oxford Centre for Clinical Magnetic Resonance Research (OCMR), Oxford University, as registrar in Clinical Physiology at Skåne University Hospital Lund, and as research fellow in the Cardiac MR Group at Lund University. As a researcher he develops and employs advanced MRI techniques to further a mechanistic understanding of cardiac function, aiming to improve the standard of care for heart failure patients.
