# Supplementary figures and images for: Non-invasive left ventricular pressure-volume loops from cardiovascular magnetic resonance imaging and brachial blood pressure: validation using pressure catheter measurements
Source: Eur Heart J Imaging Methods Pract. 2023 Oct 25;1(2):qyad035. doi: 10.1093/ehjimp/qyad035 (PMC10631830; doi:10.1093/ehjimp/qyad035)

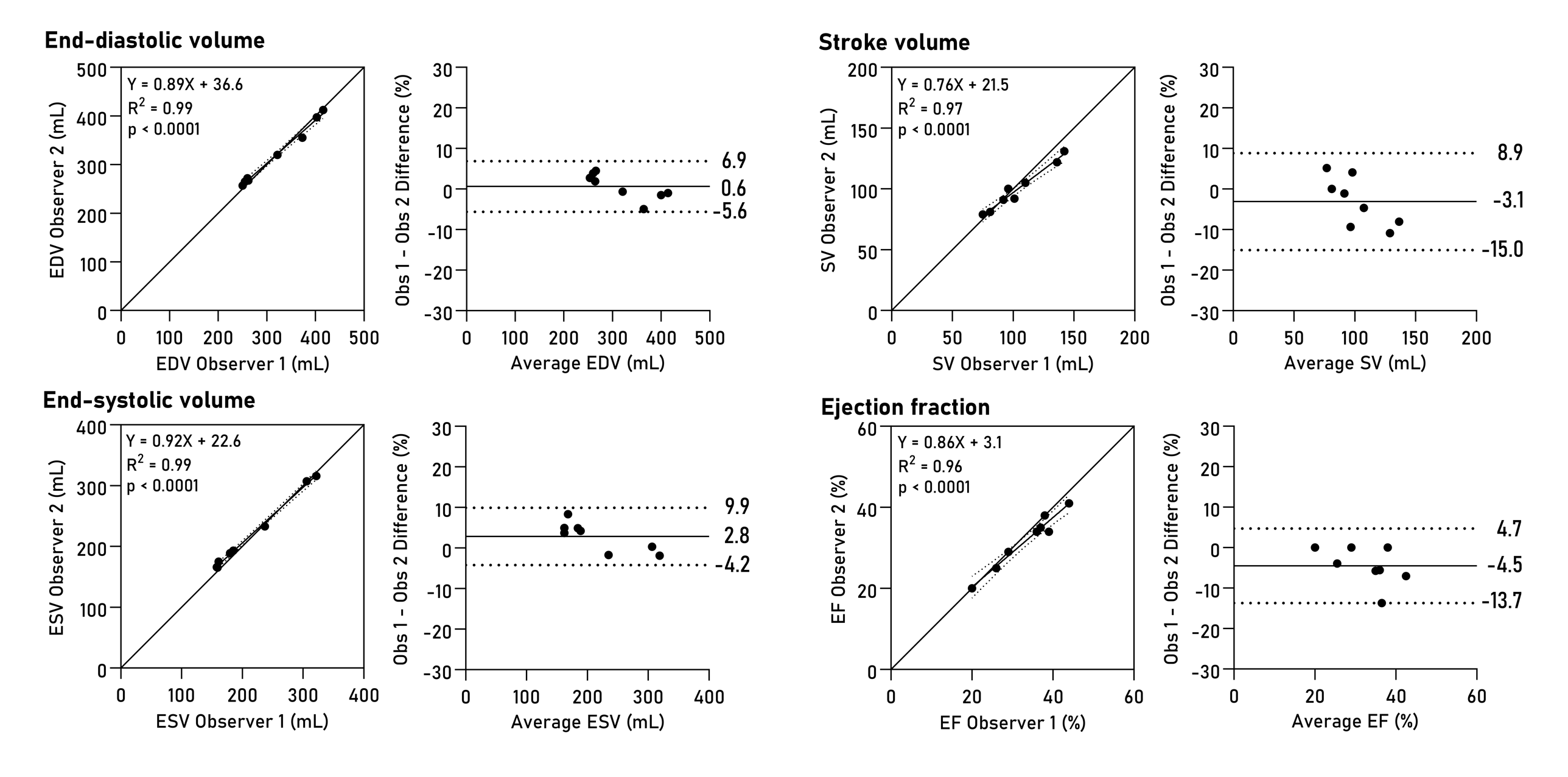

Supplement: qyad035_Supplementary_Data [file qyad035_Supplementary_Data.zip › Figure S2.png]

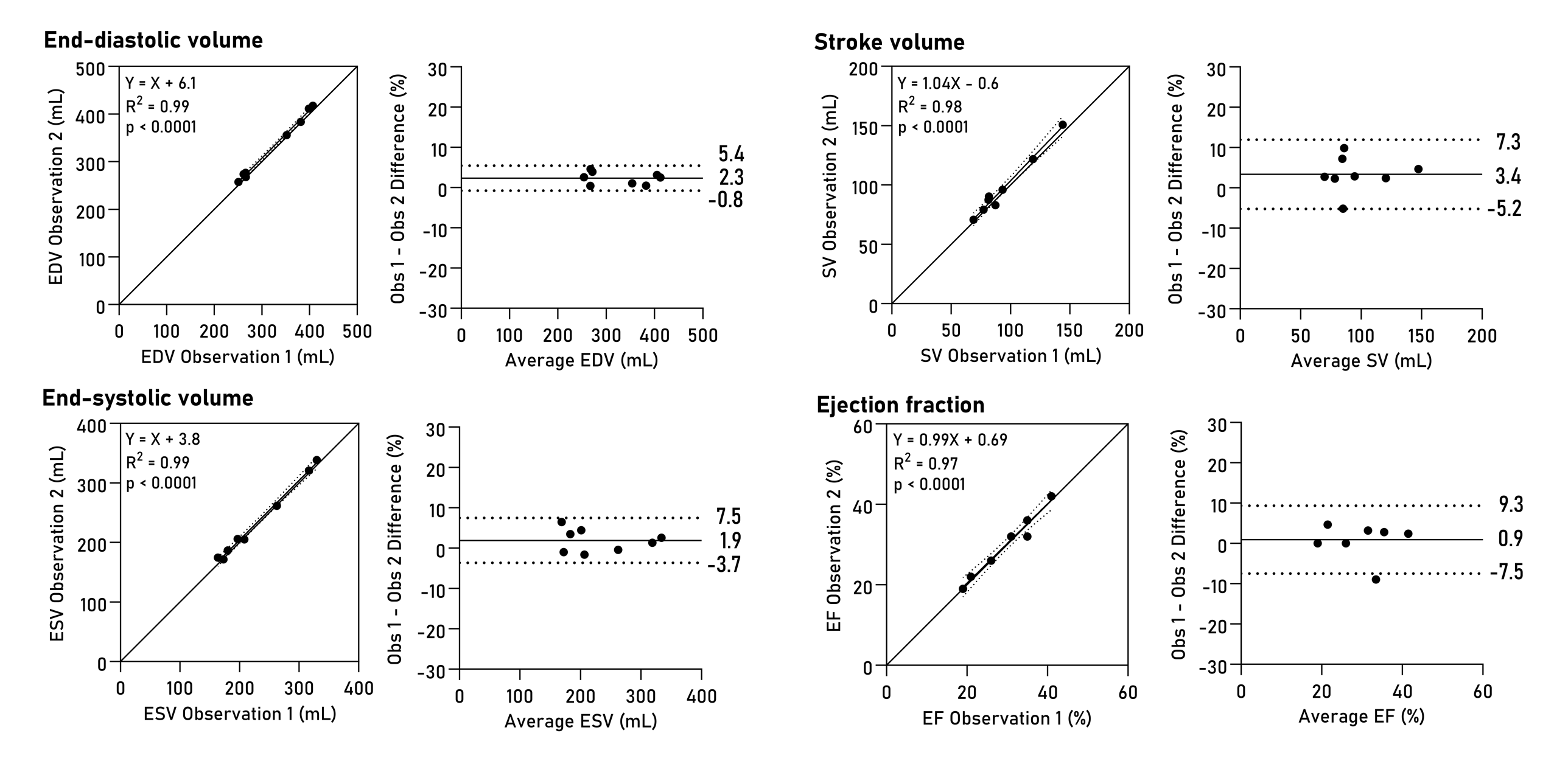

Supplement: qyad035_Supplementary_Data [file qyad035_Supplementary_Data.zip › Figure S1.png]
